# Genome-wide analysis of genetic and epigenetic control of programmed DNA deletion

**DOI:** 10.1093/nar/gku619

**Published:** 2014-07-12

**Authors:** Estienne C. Swart, Cyril Denby Wilkes, Pamela Y. Sandoval, Miroslav Arambasic, Linda Sperling, Mariusz Nowacki

**Affiliations:** 1 Institute of Cell Biology, University of Bern, Baltzerstrasse 4, 3012 Bern, Switzerland; 2 CNRS UPR3404 Centre de Génétique Moléculaire, 1 avenue de la Terrasse, Gif-sur-Yvette F-91198 cedex, France; 3 Université Paris-Sud, Département de Biologie, Orsay, F-91405, France

## Abstract

During the development of the somatic genome from the *Paramecium* germline genome the bulk of the copies of ∼45 000 unique, internal eliminated sequences (IESs) are deleted. IES targeting is facilitated by two small RNA (sRNA) classes: scnRNAs, which relay epigenetic information from the parental nucleus to the developing nucleus, and iesRNAs, which are produced and used in the developing nucleus. Why only certain IESs require sRNAs for their removal has been enigmatic. By analyzing the silencing effects of three genes: *PGM* (responsible for DNA excision), *DCL2/3* (scnRNA production) and *DCL5* (iesRNA production), we identify key properties required for IES elimination. Based on these results, we propose that, depending on the exact combination of their lengths and end bases, some IESs are less efficiently recognized or excised and have a greater requirement for targeting by scnRNAs and iesRNAs. We suggest that the variation in IES retention following silencing of *DCL2/3* is not primarily due to scnRNA density, which is comparatively uniform relative to IES retention, but rather the genetic properties of IESs. Taken together, our analyses demonstrate that in *Paramecium* the underlying genetic properties of developmentally deleted DNA sequences are essential in determining the sensitivity of these sequences to epigenetic control.

## INTRODUCTION

Early studies of mating-type inheritance in the ciliate *Paramecium* by Tracy Sonneborn (1) served as inspiration (2) for the second definition (3) of epigenetics, by the ciliate biologist David Nanney, as a way of stably transmitting different phenotypes in cells with the same genotype (4). Though Nanney recognized the importance of this type of epigenetics in development, his usage is distinct from the original usage from Waddington (5) which is more akin to what is currently referred to as developmental biology (3). Soon after Nanney's definition of epigenetics, Joshua Lederberg coined the term ‘epinucleic’ to refer to information which is expressed in a form other than the sequence of nucleotides in a nucleic acid (as ‘adjuncts’) (6). From the fusion and continued evolution of Nanney and Lederberg's definitions, we now have more modern definitions of ‘epigenetics’, such as “the study of changes in gene function that are mitotically and/or meiotically heritable and that do not entail a change in DNA sequence” (7).

Since Sonneborn's studies of mating-type inheritance in *Paramecium*, a more general form of epigenetic inheritance (8) affecting programmed DNA deletion during macronuclear genome development and known as ‘homology-dependent maternal inheritance’ (9) has been discovered in *Paramecium* (10–15) and the ciliate *Tetrahymena thermophila* (16). This type of inheritance has recently been shown to underlie *Paramecium* mating-type inheritance (17). Both the study of mating-type determination and inheritance (17) as well as earlier research (18) suggest that RNA interference-related small RNAs (RNAi-related sRNAs) known as scnRNAs (‘scan’ RNAs (19)) are necessary for this type of epigenetic inheritance in *Paramecium*. scnRNAs also appear to be the basis for epigenetic inheritance of programmed DNA deletion in *Tetrahymena* (19–21). In contrast, a different class of sRNAs, known as macRNAs (macronuclear RNAs) (22), appear to be necessary for epigenetically inherited DNA retention in the ciliate *Oxytricha trifallax* (23).

RNAi pathways rely upon a few key conserved proteins to produce sRNAs, i.e. Dicer, Piwi/Argonaute and RNA-dependent RNA polymerases, which were acquired early in eukaryotic evolution from protein domains originally involved in RNA processing and DNA repair in bacteria, archae and phages (24,25). In the eukaryotic common ancestor, these pathways may initially have served a defensive role against viruses and transposons (24), but, along with the evolutionary radiation of eukaryotes, gene duplication and protein domain shuffling have led to the diversification of these pathways, so that now the most notable role of sRNAs is in host gene regulation (24,25), including during development (26). Other than the role of sRNAs in DNA deletion/retention in ciliates, there is growing recognition of important roles for sRNAs beyond gene regulation, including in the repair of double-stranded DNA breaks in eukaryotes (27–29).

*Paramecium tetraurelia* presents an original model to study RNAi-related proteins involved in targeted DNA deletion. Many of these proteins in *Paramecium* have been generated through gene duplication and functional diversification (18,30-31) from RNAi progenitors that produce and use small interfering RNAs (siRNAs) (32–36). The ability to analyze both tens of thousands of deleted DNA regions and sRNAs that match them via high-throughput sequencing now allows us to address deeper questions about the role of genetic and epigenetic control of DNA deletion during *Paramecium*'s extensive genome reorganization, and the factors involved in this process.

Dimorphic, functionally differentiated nuclei and genomes within the same cell are quintessential ciliate characteristics. The diploid germline micronucleus (MIC) of ciliates can undergo meiosis and fertilization to transmit the genetic information to the next sexual generation, while their polyploid somatic macronucleus (MAC) contains a reorganized version of the germline genome specialized for gene expression. During *Paramecium* sexual development, a new MAC genome is generated from a reorganized duplicate of the zygotic MIC genome. In *Paramecium*, the most studied type of genome reorganization is the precise deletion of ∼45 000 internal eliminated sequences (IESs) (37). IESs are typically unique and interrupt MAC-destined coding sequences, introns and intergenic regions in the MIC genome. Precise IES elimination is essential for correct protein translation. In addition to precise DNA deletion, there is also imprecise deletion of minisatellites and transposon sequences from the MAC genome, which usually leads to chromosome fragmentation and *de novo* telomere addition (38). While the new *Paramecium* MAC genome is developing it is also amplified to ∼800N (39).

A domesticated piggyBac transposase, PiggyMac (encoded by the *PGM* gene) (40), is proposed to be responsible for both general elimination of transposon-containing MIC-specific DNA and IES excision in *Paramecium* (40). A TA dinucleotide is found at both extremities of each IES, and following excision, processing and ligation of the two 4 nucleotide 5′ overhangs a single TA is retained in the MAC DNA (41). piggyBac transposons belong to the ‘cut-and-paste’ subclass of transposons like Tc1-Mariner transposons (42), but unlike Tc1-Mariner transposons their excision is clean and does not leave a scar (43). Including the TA dinucleotide, *Paramecium* IES ends possess an ∼6 nt Tc1/mariner terminal inverted consensus (37,44).

The 45 000 *Paramecium* IESs, identified from DNA retained following PiggyMac silencing, are typically short, with a length mode of 28 bp (counting only one TA), ranging from 26 bp to over 5 kb in length (37). Consistent with the hypothesis that IESs may have originated as transposons, some IESs were shown to be their decayed remnants (37). The *Paramecium* IES length distribution has distinctive peaks every ∼10 bp (maximal at 28 bp, and declining afterward; notable up to about 140 bp in length), and a paucity of ∼38–46 bp IESs (centered around a ‘forbidden’ peak), which is thought to reflect the geometry of DNA and the co-operativity of PiggyMac during IES excision (37).

**Figure 1.**
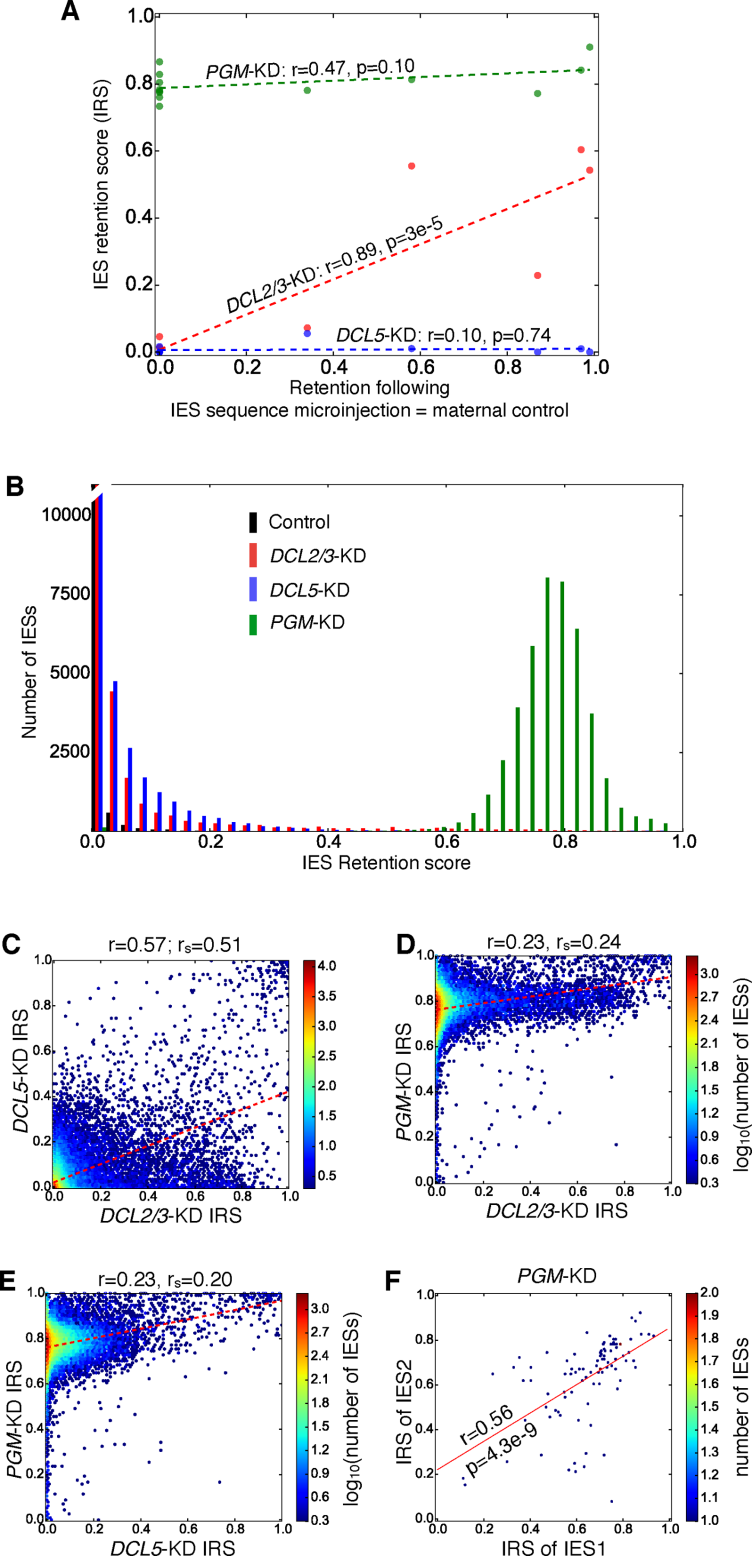
IES retention scores. IES retention scores (IRS) are the scores calculated in this paper. (**A**) To examine the relationship between maternal control and IRS, maternal control scores were calculated as the maximal retention previously observed in Figure 6 of (45), with the IRS calculated in the present study (both maternal control scores and IRS are given in Supplementary Table S1). Dashed lines corresponding to linear regressions are shown along with Pearson's r, and its two-tailed *P*-value. (**B**) IRS histograms determined for all known *P. tetraurelia* IESs for a control and knockdowns of *DCL2/3*, and *DCL5*. (**C-E**) Correlations of IRSs among knockdowns pairs of *DCL2/3*, *DCL5* and *PGM*. Pearson's r and Spearman's r (r_s_) are given above each graph. (**F**) Correlation in IRS between identical IESs. Only IESs with *PGM*-KD IRS ≥ 10% of their control IRS, and on MAC+IES scaffolds > 20 kb long were used. Pearson's r is given above the linear regression (two-tailed *P*-value < 1e-6). IESs with *PGM*-KD IRS = 0 were excluded.

**Figure 2.**
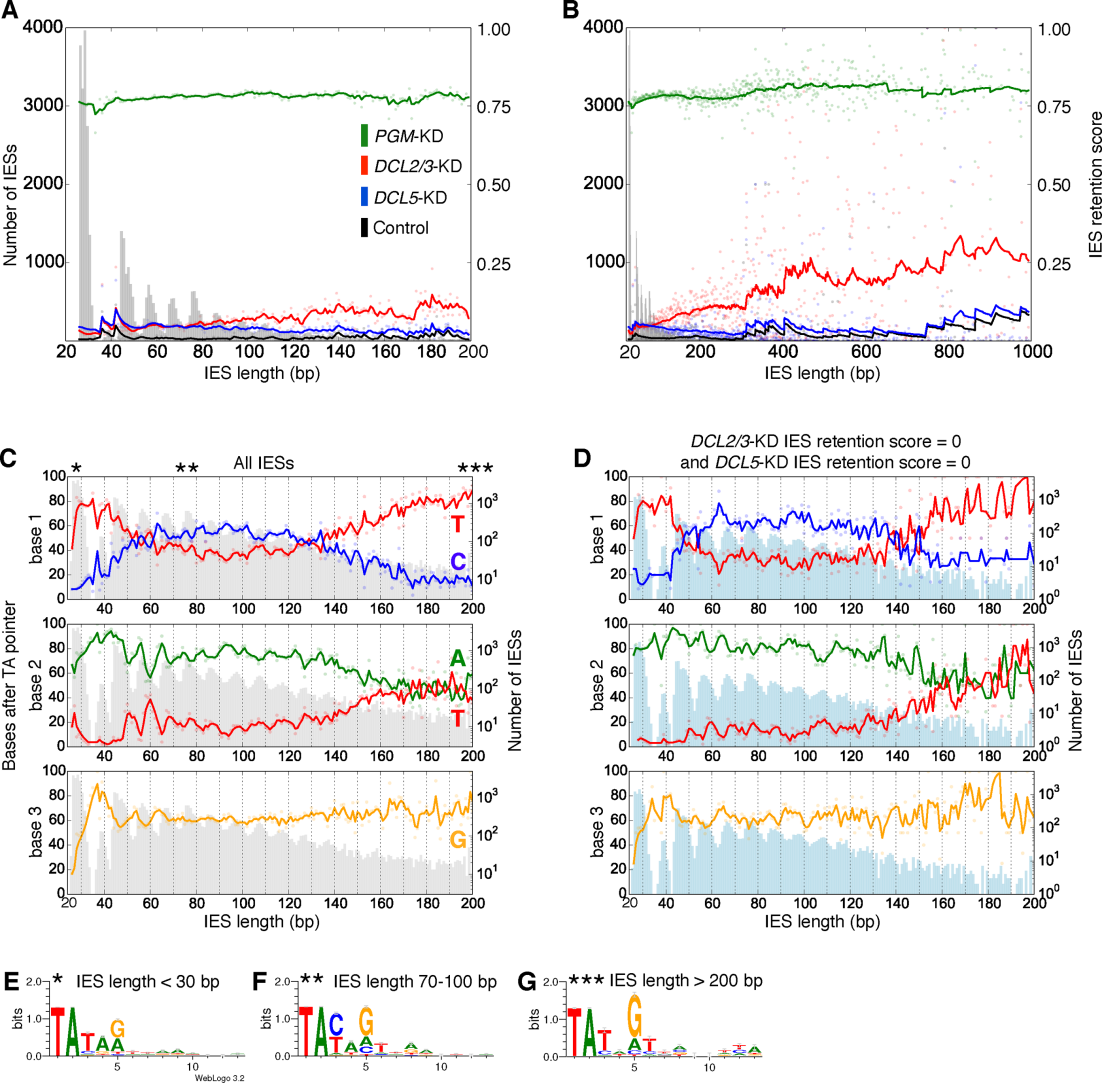
Relationships between IRSs and length, and IES length and sub-terminal base frequencies. (**A, B**) IRSs versus IES length over shorter (A) (≤ 200 bp) and longer (B) (≤ 1000 bp) IES length scales. The IES length distribution is shown in gray in the background. Lines are the exponentially-weighted mean averages (EWMA) with spans of 5 (A) and 50 (B) bp. (**C**) Base frequencies of sub-terminal positions 1–3. Only the base frequencies of the bases with the highest frequencies are shown. Solid lines are EWMAs with a span of 3 bp. The distribution of IES lengths is shown in gray behind the base frequency graphs (log_10_ scale for the right y-axis). Approximate positions of the IES length cut-offs for the sequence logos of IES termini shown in (E–G) are indicated by stars. The distribution of IES lengths of IESs with *DCL2/3*-KD IRS = 0 and *DCL5*-KD IRS = 0. The distribution of IES lengths of IESs with *DCL2/3*-KD IRS = 0 and *DCL5*-KD IRS = 0 is shown in light blue behind the base frequency graphs. (**D**) Graph as in (C), but only using IESs with both *DCL2/3*-KD IRS = 0 and *DCL5*-KD IRS = 0. (**E–G**) Sequence logos of the 13 IES bases including the TA repeat, for three ranges of IES lengths highlight the bases with substantial information content.

The first molecular evidence that the deletion of some IESs might be epigenetically controlled was obtained by microinjecting plasmid DNA including IES sequences into the maternal (old) MAC (10). Approximately a third of the IESs surveyed with this technique were retained in new MAC chromosomes after sexual development (45), and hence are known as ‘maternally controlled’ IESs (10,45) (or ‘mcIESs’; in this manuscript we specifically reserve the terms ‘maternally controlled IES’ and ‘nonmaternally controlled IES’ for the IESs tested by these experiments). In these experiments, mcIESs were retained to varying degrees depending on which IES was being examined and the amount of DNA being microinjected, and in some cases, these IESs were retained through successive sexual generations (10,45). In mutagenesis experiments, a C-to-T point substitution located three bases internal to the TA of an mcIES end completely abolished the IES's excision, suggesting that IES sequences may override maternal control of IES excision (46).

In *Paramecium* and another oligohymenophorean ciliate, *Tetrahymena thermophila*, it is now thought that the development-specific scnRNAs are necessary for targeting DNA excision (18–19,31,47-50). In *Paramecium*, scnRNAs are 25 nt long and the Dicer-like proteins Dcl2 and Dcl3 generated their dsRNA precursors at meiosis from dsRNA transcribed across the entire MIC genome (18,31). Before being transported to the old MAC, double-stranded scnRNAs have been proposed to be bound to two Piwi-like proteins (Ptiwi01 and Ptiwi09) (30), resulting in cleavage and removal of their passenger strands (31) (as has been demonstrated for *Tetrahymena* scnRNAs and the *Tetrahymena* Piwi-like protein Twi1p (51)). In both *Tetrahymena* and *Paramecium*, old MAC scnRNAs complementary to this genome are subtracted by a process known as RNA scanning (18–19,48,49,^52^). The remaining scnRNAs are transported to the developing new MAC where they target DNA elimination by PiggyMac in *Paramecium*, or by the PiggyBac homolog, Tpb2p (53) in *Tetrahymena* (models for RNA scanning and DNA targeting by scnRNAs in *Paramecium* and *Tetrahymena* are reviewed in (54,55)). scnRNAs may therefore be considered a primary transport of epigenetic information for DNA deletion from the old to the new MAC. As judged by PCR analyses, cosilencing of the *DCL2/3* genes led to observable retention of five of seven mcIESs and zero of seven non-mcIESs, while more sensitive DNA-seq analyses showed retention of all these mcIESs and weaker retention (<5%) of two of the seven non-mcIESs (18). Overall, 1/9th of all *Paramecium* IESs showed 5% or more retention in DNA-seq data following *DCL2/3* cosilencing (18).

Recent work has identified a second class of development-specific sRNAs (iesRNAs) in *Paramecium*, which are ∼21–31 nt long (mode of 27 nt) (18). Dcl5, a distinct Dicer-like protein which is responsible for producing the dsRNA precursors of iesRNAs, localises specifically to the new developing MAC. In contrast to scnRNAs, which overlap IES boundaries into surrounding non-IES sequences, iesRNAs precisely match within IESs and have a more pronounced tendency to accumulate at IES ends, suggesting they are produced from transcripts of excised IESs (18). iesRNAs were proposed to serve a general role in ensuring complete IES excision (18). This role differs from that of scnRNAs, which become increasingly important for IES removal as IES length increases and are essential for the removal of transposons located in imprecisely eliminated MIC-specific genomic regions (18). Since iesRNAs are produced in the developing new MAC they are not a primary transport of epigenetic information like scnRNAs, and indeed, as assessed by PCR the silencing of *DCL5* only appears to affect some mcIESs—two of seven mcIESs versus zero of seven non-mcIESs; however, four additional mcIESs and two additional non-mcIESs appear to be weakly affected (<5% retention) using DNA-seq data (18). For some IESs, iesRNAs may thus provide a complementary source of targeting that does not require epigenetic information. On the other hand, iesRNAs may serve as a secondary transport of epigenetic information for IESs requiring scnRNA targeting if their production depends on IES excision (18).

While our understanding of the mechanisms involved in scnRNA production continues to improve, it has been unclear precisely what determines the relationship between scnRNAs and their necessity in IES targeting, or why excision of some IESs is more strongly epigenetically controlled than others. To address these problems, we have scrutinized the relationship between scnRNAs, iesRNAs and DNA excision using genome-wide analyses of IES retention and sRNA populations from *PGM, DCL2*/*DCL3* and *DCL5* knockdowns. We show that relative to IES length, there are pronounced differences in the IES sub-terminal base frequencies, suggesting that the IES excisase's recognition/excision preferences change with IES length. We also show that the sub-terminal base frequencies of IESs most requiring scnRNAs for complete deletion have different biases from those least requiring them. For complete IES deletion, the same trends were observed for IESs most requiring iesRNAs versus those least requiring them. This suggests a model where some IESs are more difficult to recognize and/or excise than others, with those that are most difficult to recognize/excise consequently requiring the greatest targeting support from scnRNAs and iesRNAs.

## MATERIAL AND METHODS

### *Paramecium* strains, cultivation and autogamy

All experiments were carried out with *P. tetraurelia* strain 51. Cultivation and autogamy were carried out at 27°C as previously described (18,45).

### Gene silencing and preparation of libraries for Illumina DNA-seq and sRNA-seq

Gene silencing of *DCL2/3* and *DCL5* was carried out by RNA interference, as previously described (18).

For the *DCL2/3* and *DCL5* knockdowns, macronuclear DNA was isolated from postautogamous cells starved for 5 days until the bulk of the old MAC fragments were degraded leaving primarily new macronuclei, after which standard paired-end Illumina libraries were prepared for DNA-seq (Experiment 4 of (18); deposited together under the NCBI short read archive (SRA) accession: SRX387766). The generation of the control DNA (NCBI SRA Accession: ERX114454) and *PGM*-KD (accession: ERX114957 and ERX114955) DNA-seq data sets was previously reported in (37).

In a separate experiment (Experiment 2 of (18)), RNA isolations and small RNA libraries were produced and sequenced according to standard Illumina protocols for control, *DCL2/3*-KD and *DCL5*-KD cells, with only the ‘late’ developmental time point used in the present study (deposited under accessions SRR907875, SRR907877 and SRR907883, respectively).

### Reference genomes

The following reference genomes were used in the IES analyses and were used for read mapping:

MAC: http://paramecium.cgm.cnrs-gif.fr/download/fasta/ptetraurelia_mac_51.fa
MAC+IES: http://paramecium.cgm.cnrs-gif.fr/download/fasta/ptetraurelia_mac_51_with_ies.fa

### Determination of genome-wide IES retention scores (IRSs)

After quality filtering and removal of adapters, Illumina reads were mapped to the reference genomes (*P. tetraurelia* MAC reference genome and MAC+IES reference genome) using BWA (56), and Samtools (57) for indexing.

For each sample, IES retention scores (IRSs) were determined for each IES in the genome by counting the number of reads that contain the IES sequence (symbolized IES^+^) and the number of reads that contain only the macronuclear IES junction comprising a TA dinucleotide (IES^−^). Only read pairs which mapped unambiguously were counted. Each read was counted only once to avoid overcounting owing to paralogous matches. Reads were only counted at IES ends, to avoid length biases resulting from IES length variation. The retention score of an IES is then given by the equation: IRS = IES^+^/(IES^+^ + IES^−^). All the IRSs may be obtained via ParameciumDB (58) and are also provided in the Supplementary Data file online (IES_retention_scores.txt).

### TA-indel analysis

Paired-end mapping to the reference MAC genome was used to find reads containing a deletion between 5 bp and 10 kb in length. A custom Perl script was then used to realign the ends of the alignment between the deleted segment and the reference. Deletions bound by TA dinucleotides in the reads were counted, except when they contained ‘N’ in the deleted sequence. For each dataset, the normalization is the ratio of reads with TA-indels per million mapped reads.

As a validation of this method, we found very similar results using Illumina sequencing reads of the same DNA sample previously used to analyse TA-indels in Sanger reads (59) (data not shown).

### sRNA-seq mapping

When necessary, Illumina sequencing adaptors were removed from the reads, and reads which matched known contaminants (bacterial DNA, rDNA, mitochondrial DNA, plasmid DNA) were discarded. Reads were separated into different size classes (15–35 nt) and mapped with BWA (version 0.6.2-r126) (56) using the default parameters, with the exception of the maximum differences parameter, which was set to ‘-n 0’ to select exact matches. Mapped reads were then filtered with custom Perl or Python scripts to select reads matching unique locations in each of the data sets. We mapped the data to the whole MAC genome (*P*. *tetraurelia* 51 strain) and then those reads that do not match were mapped to the MAC+IES genome. Read counts were normalized to the total number of 15 and 35 nt long sRNAs mapped to the MAC and the MAC+IES reference genomes in each sample (this normalization is similar in principle to the one we previously used (18)).

### Sequence logos

Sequence logos were created with WebLogo (60), version 3.2 or 3.3, and were normalized to the base frequencies of internal IES positions > 10 bp from the TA repeat (A = T = 0.4 and C = G = 0.1), since the background base frequencies are likely to be those in DNA that are closest to evolving neutrally.

## RESULTS

### The effects of silencing of *DCL2/3*, and *DCL5* on IES excision

Previously, IES retention following gene silencing was typically assayed (e.g. in (61)) for the small subset of seven maternally and eight nonmaternally controlled IESs (see Supplementary Table S1). To gain a clearer picture of the effects of silencing of *DCL2/3* and *DCL5* on IES excision, we analyzed IES retention in high-throughput MAC DNA sequence data. For each IES, we define an IRS as IRS = IES^+^/(IES^+^ + IES^−^), counting reads mapping to the IES as IES^+^, and reads that map across the spliced junction of the IES as IES^−^ (see Materials and Methods). Thus, a completely excised IES has a score of 0, and a completely retained IES has a score of 1. For the experimentally determined subset of mc- and non-mcIESs (Figure 1A; Supplementary Table S1) it can be seen that IRSs are positively correlated with maternal control scores for the knockdowns of *DCL2/3*. For *PGM*-KD and *DCL5*-KD, the correlation coefficients are not statistically significant at α = 0.05, precluding drawing conclusions about possible correlations between retention scores and maternal control. Clearly, due to the small number of mc and non-mcIESs, there is a need to inspect IES retention more generally.

For all the known *P. tetraurelia* IESs (in the ‘MAC+IES assembly’; see methods) IRS histograms for the different gene silencings and control considered in this paper are shown in Figure 1B. We found that IRS estimates are reproducible for a pair of biological replicates of a different *Paramecium* gene involved in developmental DNA deletion (Spearman correlation coefficient = 0.92, *P* < 2.2 × 10^−16^; (Swart, Denby Wilkes, in preparation)). The control histogram shows the lowest levels of IES retention, and the *PGM*-KD histogram shows the most severe IES retention. As we show later (see ‘Gene silencing reveals new properties of eliminated DNA in *Paramecium*’), at least some of the variance in the *PGM*-KD IES retention appears to be due to natural variation in the efficiency of excision by PiggyMac. The histograms of *DCL2/3*-KD and *DCL5*-KD IRS are both right-skewed, i.e. most copies of the IESs are properly excised for the majority of IESs affected by these silencings. Weak to modest positive correlations can be seen among the IRSs following the *DCL2/3*, *DCL5* and *PGM* silencings (Figure 1C–E), suggesting that there may be some indirect associations between scnRNAs, iesRNAs and the PiggyMac excisase complex.

Though almost all *P. tetraurelia* IESs are unique, a small number of IESs are identical (37), providing the opportunity to examine IESs with the same internal genetic constraints. The retention scores of identical IESs are moderately correlated, suggesting that IRSs have a significant genetic component (Figure 1F; note that these IESs have one or more differences in their flanking regions to discriminate between their IES^+^ reads). Since identical IESs may be generated by tandem duplications, we considered the possibility that their proximity to each other might affect their retention scores. Of the 93 identical pairs of IESs in the *PGM*-KD in Figure 1F, 54 had IESs on different scaffolds, and for the remaining 39 the mean distance between the identical IESs was 27.8 kb. Thus we can exclude the possibility of close proximity as a possible reason for the observed correlations in retention scores of the identical IESs. The observed variation in retention scores between identical IESs might then either be due to genetic differences in their flanking sequences, some inaccuracy in our measurement of retention scores, or due to genuine differences in epigenetic control of these IESs.

To examine the possibility of proximity effects on retention scores, we examined correlations between retention scores of pairs of adjacent IESs (Supplementary Figure S1A–C). For the *DCL2/3*- and *DCL5*- knockdowns, the correlation between these IESs was negligible (*r* < 0.12; Supplementary Figure S1A–C). However, for the *PGM*-KD, a weak positive correlation of IRSs between adjacent IESs is evident (*r* = 0.27, two-tailed *P*-value < 1 × 10^−45^). This correlation between *PGM*-KD IRSs of pairs of adjacent IESs increases when the distance between the adjacent IESs decreases (e.g. *r* = 0.45 with < 200 bp of intervening sequence; Supplementary Figure S1E), and decreases to negligible levels over longer scales, and with more intervening IESs (e.g. *r* = 0.08 for > 10 kb between 10 IESs; Supplementary Figure S1D; no correlation (*r* = 0) is observed between random IESs - Supplementary Figure S1F). In general for all the IRSs the closer a pair of IESs are together, the stronger the association of their IRSs (Supplementary Figure S1G and S1H). This suggests either some interaction between IESs, or the influence of factors that span multiple IESs, such as chromatin state.

### Gene silencing reveals new properties of eliminated DNA in *Paramecium*

To examine the relationship between the genetic and epigenetic determinants of IES excision in *Paramecium* on a genomic scale, we scrutinized differences in basic properties of IESs with respect to their retention following the silencing of *DCL2/3, DCL5* and *PGM*.

**Figure 3.**
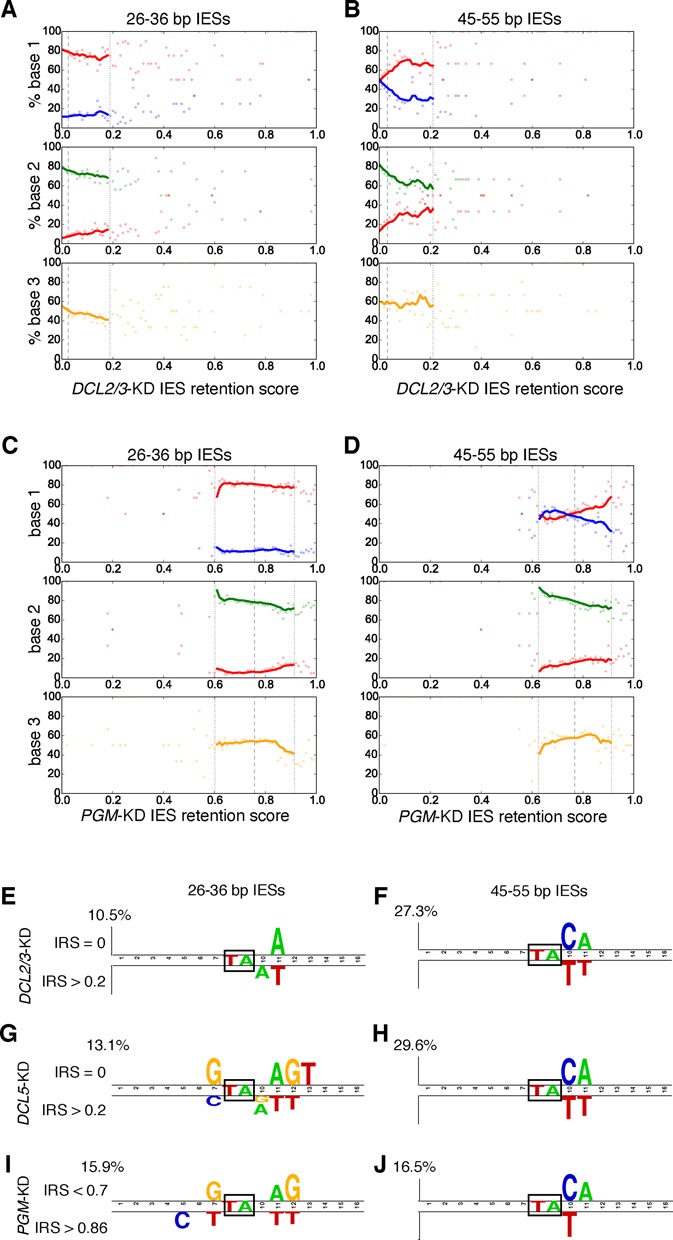
Relationships between IRSs and sub-terminal base frequencies. (**A–D**) Base frequencies of sub-terminal positions 1–3 compared to the IRS of *DCL2/3*-KD (A and B) and *PGM*-KD (C and D), for IESs from the first (26–36 bp) and third (45–55 bp) length peak. Only 45–55 bp IESs are shown, but similar trends are visible for ∼10 bp windows surrounding longer IES length peaks. EWMA lines for spans of 10 data points (intervals of 0.01) are only plotted for data within two standard deviations (dotted vertical lines) of the mean retention score (dashed vertical line). Mean IES length across the range of retention scores remains essentially constant (e.g. Supplementary Figure S2A). Also see Supplementary Figure S2B–E for graphs of IES end base frequency versus IES length for the *DCL5*-KD and the control. **(E–J)** Two sample sequence logos for the region surrounding boxed IES TA repeats (‘MAC’ = MAC destined sequences) showing bases that are enriched in either non-retained IESs (above) or moderately to strongly retained IESs (below). Only statistically significant bases with a Bonferroni-corrected *P*-value < 0.0001 (*t*-test) are shown (E–I) and a Bonferroni-corrected *P*-value < 0.05 (*t*-test) for (J). Two sample logos for IESs from the first (26–36 bp), and third (44–54) IES length peaks are shown, but similar logos are produced for subsequent length peaks. Note that the ordinate axes differ in scale (percentages given above the axes).

Previously we showed that in *DCL2/3*-KD cells, average IES retention increases with IES length, while IES retention in *DCL5*-KD cells remains relatively constant (18) (also shown in Figure 2A and B with the IRSs determined in the present paper, rather than the cruder estimates previously determined (18)). We also found intriguing relationships between the bases present at the ends of IESs, IES length and IRSs (Figures 2 and 3). Since the first five bases of IES ends have the most information content, and the outer TA is invariant, we concentrated our analysis on the next three inner bases (i.e. the sub-terminal bases). Figure 2C shows that these sub-terminal base frequencies, particularly for positions 1 and 2, change substantially with IES length. For the shortest IESs, corresponding to the first peak of the IES length distribution, a T is almost always preferred at the 1st base. For IES length between the 4th peak and about 135 bp, C is marginally the preferred nucleotide, while for larger IESs, T is again preferred. At the 2nd base, A is almost always preferred, however for IESs larger than about 150 bp, A and T are equally likely. At the 3rd base, G is the preferred nucleotide. There is a very dramatic increase in G frequency from less than 20% to greater than 90% from the shortest IESs to the ‘forbidden’ 2nd peak. Substantial differences in base composition of IES ends for different IES length ranges can also be visualized by sequence logos, which highlight that just the first few IES positions following the TA have constrained base frequencies (Figure 2E–G).

For the first five IES length peaks (∼28–57 bp, including the ‘forbidden’ peak) in Figure 2C smaller scale fluctuations in base frequencies of opposing phase to IES lengths are visible for bases 2 and 3 (i.e. IES length troughs correspond to base frequency peaks and vice versa). The trends in base frequency changes over both longer and shorter scales (hundreds and tens of bp, respectively) are similar for IESs with *DCL2/3*-KD and *DCL5-*KD IRSs of zero (Figure 2D). This suggests that the recognition/excision efficiency of IESs not requiring, or with a much lesser sRNA targeting requirement, also varies with their sub-terminal bases and lengths. Since PiggyMac is a member of the PiggyBac clade of transposases, it is possible that other PiggyBac transposases might have similiar dependencies between transposon length and the terminal base frequencies of the transposons they excise.

To examine the relationship between base frequencies and IRSs without the confounding effects of IES length, we negated the positive association between IES length and retention score by measuring base frequencies for IESs in ∼10 bp windows surrounding the first and third IES length peaks (Figure 3; that this negation is effective is shown in Supplementary Figure S2A for *PGM*-KD as an example). For the *DCL2/3*-KD, with the exception of the most abundant and short IESs (26–36 bp; Figure 3A) the first sub-terminal position's T frequency increases and then plateaus with IRS (mirrored by the C frequency), while the second position's A frequency decreases with IRS (mirrored by a T-frequency increase). Similar base frequency changes are visible in the *DCL5*-KD (Supplementary Figure S2B and C). Two sample sequence logos (62) for the first and third IES length peaks for two extremes of IRSs show that the bases most affected by the silencing of *DCL2/3, DCL5* and *PGM* in long IESs are the two sub-terminal bases (Figure 3F, H, J). For the shortest IESs (Figure 3E, I, J,) there also appear to be smaller, but similar differences in the base frequencies of positions around the TA repeat. A notable difference for the smaller IESs is the non-IES position immediately before the TA repeat, which is enriched in G in two of the three knockdowns. This base is included in excised IESs, which have four base 5′ overhangs centered around their TA (41).

It should be noted that, for the sake of simplicity, we have examined just one of the two IES ends while analyzing the effects of gene silencing on IESs; however, both ends appear to influence the IRS. For instance, for longer IESs (> 45 bp), IESs with two TT sub-termini generally have higher *DCL2/3*-KD IRSs (mean 0.10) than IESs with both a TT and a CA sub-terminus (mean 0.06), which are in turn higher than the retention scores of IESs with two CA sub-termini (mean 0.04; Supplementary Figure S2F).

### The effects of silencing of *DCL2/3* and *DCL5* on aberrant DNA excision

**Figure 4.**
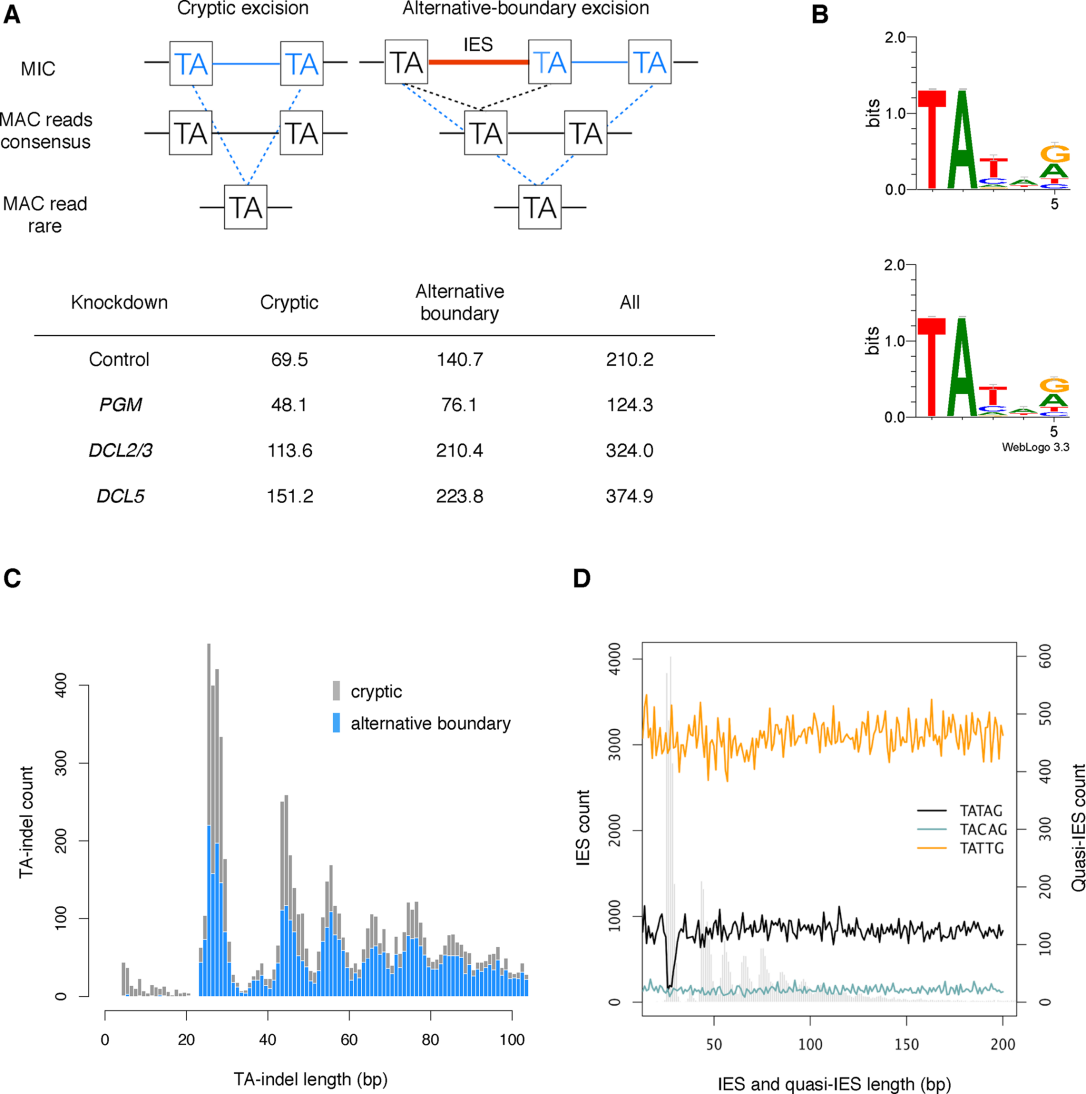
Effects of gene silencing on DNA excision errors. (**A**) Two kinds of low-frequency TA-indels are shown schematically, with the deletion shown in blue. In the ‘cryptic excision’ TA-indel class MAC-destined DNA is excised, while in the ‘alternative-boundary’ class an IES is excised beyond its usual boundary into the flanking MAC-destined DNA. The table shows the number of reads per million for each class of TA-indels. (**B**) Sequence logos for low-frequency TA-indels in sequence data from the control (upper) and the *DCL2/3*-KD (lower). (**C**) Histogram of cryptic (gray) and alternative-boundary (blue) TA-indels in the *DCL2/3-*KD sequence data. Both types of TA-indels have a periodic distribution resembling the IES length distribution, dominated by the first peak, which is consistent with the observed constraint on IES length (37). (**D**) The reference MAC genome was scanned for segments (quasi-IESs) flanked by different IES terminal inverted repeats: TATAG most frequently found for the shortest IESs (black curve), TACAG most frequent for IESs larger than circa 40 nucleotides (blue curve) and TATTG, most frequent for IESs with a *DCL2/3*-KD IRS > 0.2 (orange curve). Only perfectly inverted repeats were considered. The curves represent the count of the quasi-IESs for each length (counting only one of the flanking TA dinucleotides). The histogram of IES length is shown in gray in the background.

Although IES excision in *Paramecium* is relatively precise, errors do occur naturally. In wild-type strains of *P*. *tetraurelia* (37,59) and other *Paramecium* species (63), three types of low-frequency errors (‘TA-indels’) were observed: (i) occasional IES retention (‘residual TA-indels’); (ii) IESs with improper alternative boundaries and (iii) cryptic excision of sequences weakly resembling IESs (bounded by TA). All these different types of ‘TA-indels’ (Figure 4A) are thought to be produced by the IES excision machinery since their length distribution and end sequences are similar to those of IESs (37,59). Since depletion of the proteins examined in this study led to retention of some IESs, we wondered whether there might also be effects on the low-frequency deletions.

In principle, as for IES excision, both genetic and epigenetic factors may contribute to TA-indels, and so we were interested to see the effect of the knockdowns examined in this paper on these errors. Figure 4A shows the number of reads per million in each sample that contain TA-indels. To put this in perspective, cryptic excision would result in a TA-indel rate of 1.4 × 10^−6^ per site per generation in the control. These TA-indel rates are much higher than the indel rate of 4 × 10^−12^ per site per generation in *P. tetraurelia* (64) and approaching known epimutation rates, which are generally predicted to be higher than genetic mutation rates (65) (e.g. 4.5 × 10^−4^ for the CG methylation epimutation rate per CG site in *Arabidopsis* (66)). It should be noted that these rare events may be of little consequence since they are scattered throughout the polyploid macronuclear genome, and so are unlikely to influence individual genes. Both cryptic IES excision and the use of alternative boundaries are reduced in *PGM*-KD cells, in agreement with the hypothesis that TA-indels are produced by excision errors. In contrast, increased TA-indel rates in the *DCL2/3* and *DCL5*-KD samples compared to the control suggest that interfering with the sRNA-dependent IES targeting machinery results in increased erroneous excision.

Sequence logos for the low-frequency TA-indels in all of the samples show a weak preference for a 5′-TATNR-3′ consensus at the ends of the excised segments, as shown for the control and *DCL2/3*-KD TA-indels (Figure 4B). With the exception of the second base after the TA (‘N’), this consensus resembles that of the shortest IESs (Figure 2E). Moreover, the cryptic TA-indel length distribution shows a prominent peak centered around 26–30 nt (Figure 4C), corresponding to the length of the smallest IESs (as in (63)). As we previously indicated, the preferred end sequence of shorter IESs may be TATAG. Since our experiments suggest that neither scnRNAs nor iesRNAs may be required for excising many IESs (18), we wondered what prevents the IES excisase from recognizing such ends scattered throughout the DNA destined to become the MAC genome. We scanned for segments of the MAC genome bound (‘quasi-IESs’, which have the potential to be excised as cryptic IESs) by the ‘optimal’ TATAG inverted repeats, as well as segments bound by TATAC, the end consensus for larger IESs, and TATTG, the end consensus IESs most affected by the *DCL2/3* and *DCL5 1* knockdowns. The result (Figure 4D) is quite striking: TATAG quasi-IESs of the size of the first peak in the IES length distribution are present at ∼1/5th the expected number (surrounding this peak), suggesting evolutionary counter-selection against such segments. In the MAC genome, we calculated that TATAG quasi-IESs of 26–30 nt have an excision rate of 22.1 × 10 × 10^−4^, whereas the excised TACAG frequency is almost three times lower (7.62 × 10 × 10^−4^) and excised TATTG quasi-IESs have never been observed. This supports the idea that the IES excisase is able to cleave optimal short DNA segments around 26–30 nt long with little or no additional targeting information.

### Relationships between IRSs and sRNA densities

**Figure 5.**
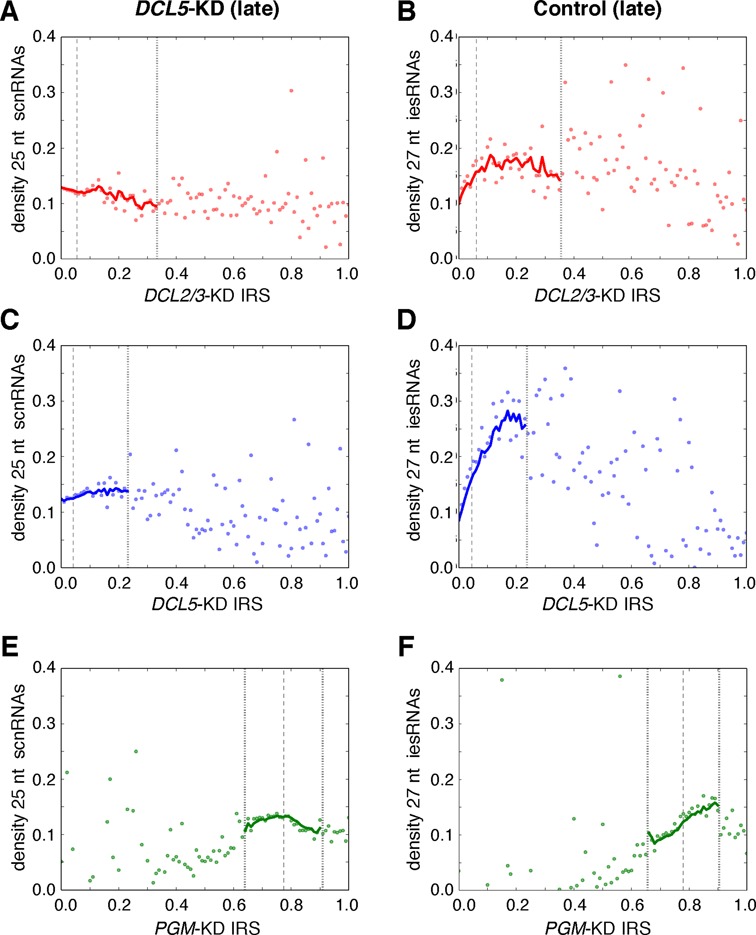
Effects of gene silencing on sRNA densities. (**A–I**) Headings above the subgraphs indicate the experimental condition or control, along with any additional constraints, and continue down the subgraph columns until the next heading. Densities of IES-matching 25 nt scnRNAs and 27 nt iesRNAs (sRNAs/base) are the median values over IES retention intervals of 0.01 in the *DCL5*-KD during late development (first column - A, C, E) and the control, late development experiment (second column - B, D, F). EWMA lines for spans of five data points (intervals of 0.01) are plotted for data within two standard deviations (dotted vertical lines) of the mean IRS (dashed vertical line) of the analyzed subset of IESs; since data outside two standard deviations are very limited and not very accurate, trend lines beyond these intervals are not shown. A few isolated sRNA density outliers (> 0.4), outside the two standard deviation intervals, are not shown for some of the subgraphs. See Supplementary Figure S3 for Control (late) 25 nt sRNA densities (a mixture of scnRNAs and iesRNAs) versus retention scores.

Since scnRNA and iesRNA quantities are affected by the silencing of *DCL2/3* and *DCL5*, we wondered if a quantitative relationship between the amount of these sRNAs and IES retention exists. We therefore examined the density of 25 and 27 nt sRNAs mapping to the IESs in control and gene-silenced cells compared to IRSs (Figure 5 and Supplementary Figure S3). As we previously showed, the 25 nt sRNAs in our control samples are a mixture of both scnRNAs and some 25 nt iesRNAs (18), but it is possible to examine scnRNAs with a minimal iesRNA contribution by examining 25 nt sRNAs from the *DCL5*-KD (18) (Figure 5A, C, E, G). In the *DCL5*-KD, it can be seen that scnRNA densities are more or less constant relative to the IRSs of all the knockdowns we examined (Figure 5A, C, E). This is consistent with uniform production of scnRNAs across the entire germline genome and little if any modification of the IES-matching scnRNA population between scnRNA biogenesis, RNA scanning and active IES excision. Furthermore, we observe the same overall 25 nt sRNA density in control samples for IESs showing no retention after *DCL2/3* cosilencing (data not shown). IESs are therefore equally likely to be covered by scnRNAs irrespective of whether they require scnRNAs for their targeting and excision.

In contrast to the situation for scnRNA densities, we observe a positive association between iesRNA density (represented by 27 nt sRNAs) and all the IRSs we examined (Figure 5B, D, F). The strongest relationship between iesRNA densities and IRSs is for the *DCL5*-KD IRSs (Figure 5D), i.e. IESs most sensitive to iesRNA depletion normally have the highest densities of iesRNAs.

## DISCUSSION

### Why is excision of some IESs more epigenetically controlled than others?

In his paper, ‘Metaphor and Mechanism: “Epigenetic Control Systems” Reconsidered’ (67), Nanney notes that concepts from cybernetics (68) and computer science (69) were borrowed and adapted in the field of epigenetics. Influence of these ideas can clearly be seen in the use of the word ‘information’ in the writing at the time (e.g. see Lederberg (6)). Our current understanding of epigenetic control of DNA deletion in *Paramecium* can also best be described in terms of information. In *Paramecium*, as the experiments showing maternal control of some IESs (mcIESs) demonstrate (10,45), the old MAC genome is a cache (in the computer science sense of being rapidly accessible and requiring renewal) of epigenetic information. This information is ‘epigenetic’ in Nanney's strict sense of being heritable information not contained within the germline DNA (4). The MAC genome may therefore be considered as an epigenome (70), while the ‘primary genetic material’ (as Nanney put it (4)) is the micronuclear genome. Before RNA scanning, by virtue of being produced from transcripts across the entire micronuclear genome, scnRNAs cannot be used to discriminate DNA to be retained from that which should not, and hence are effectively informationless. During scanning, the scnRNAs acquire epigenetic information through a filtering sequence comparison, which they then transmit to the new macronucleus, thereby becoming a central component of an ‘epigenetic control system’ (in Nanney's sense) for IES targeting and excision. Co-silencing of the *DCL2* and *DCL3* genes suggests that scnRNAs are necessary for the reproducible removal of mcIESs, but not non-mcIESs (18). We have therefore referred to the IESs whose elimination depends on the transmission of epigenetic information by scnRNAs, including the subset of mcIESs, as ‘epigenetically controlled IESs’ (or epiIESs, as in (18)).

While Nanney considered epigenetic control systems to be crucial for development, he also acknowledged that these systems rely upon genetic systems. However, he struggled with the problem of how to unambiguously distinguish between genetic and epigenetic systems, to which he did not find a satisfactory solution (4). In the present study in an attempt to get a better understanding of what determines why the deletion of some IESs is more epigenetically controlled, we began by investigating the effects of co-silencing *DCL2/3* on DNA deletion, since the Dicer-like proteins encoded by these genes were shown to be involved in the production of the sRNAs targeting epigenetically controlled IESs. In the end, we realized that the solution to this problem may lie in understanding its converse: why some IES excision is more genetically determined.

We found two notable, associated differences in genetic properties of IESs—their length and end sequences—which, we suggest, together lead to differences in the ability of IESs to be recognized/excised. The important role of genetics in IES excision can be seen in a number of ways: (i) the conservation of nucleotides at IES ends seen in sequence logos, particularly the TA dinucleotide and the next three internal IES bases; (ii) a single base substitution three bases in from the TA of an mcIES end completely overrides its excision (46); (iii) the existence of nonmaternally controlled IESs and IESs whose excision appears to be unaffected by scnRNA depletion (as judged from *DCL2/3* silencing (18)); (iv) the correlated retention of identical IESs (Figure 1E); (v) the remarkable IES length distribution (37) is associated with equally remarkable changes in base frequency, both over a longer (hundreds of bases) and a shorter scale (∼10 bp; Figure 2C); (vi) the strong selection against quasi-IESs, with lengths and end bases of ‘optimal’ (the most abundant) IESs, in DNA that becomes and is part of the macronuclear genome.

Examination of the sub-terminal base frequencies versus length of IESs with *DCL2/3*-KD and *DCL5*-KD IRSs of 0 (Figure 2D), i.e. IESs which appear to have much less or no scnRNA or iesRNA requirement for their excision, revealed both longer and shorter range base frequency trends similar to that of IESs in general, suggesting that the IES excisase complex itself dictates some of the base frequency differences between IESs. A possible explanation for the opposing long- and short-range trends in preferred base may lie in the activity of the excisase, i.e. for suboptimal IES lengths (troughs) the specific bases close to IES ends become more important for their efficient targeting/excision, whereas for optimal sequence lengths these bases are less critical. This would also explain the increase in base frequency observed for positions 2 and 3 for the 2nd IES length peak, which can be thought of as being in a major IES length trough. From this it can be predicted that short IESs with the least optimal IES length are associated with the most optimal IES end sequences, i.e. ends starting with ‘TATAG’. The same optimal end sequence is the most strongly avoided in quasi-IESs of optimal sequence lengths.

The key implication of these genetic differences between IESs is that those IESs which are not as efficiently recognized and removed, i.e. IESs with suboptimal end sequences and lengths, require additional support for complete removal of all their copies, whereas optimal IESs do not. The targeting information may either come in the form of scnRNAs (for epiIESs) or iesRNAs (any suboptimal IESs which are transcribed and produce iesRNAs). However, scnRNAs are much more important for the recognition of longer IESs, including those which are clearly transposon relicts, than iesRNAs (18).

### What is the relationship between scnRNAs, iesRNAs, IES retention and epigenetic control of IES excision?

IRSs reflect the sensitivity of IESs to gene silencing and may be thought of as having both an epigenetic control component and a genetic control component, the relative contribution of which depends on the gene/s being silenced. While it is reasonably clear that the *DCL2/3*-KD IRSs may reflect some degree of epigenetic control, since the depleted proteins are involved in scnRNA production, the *DCL5*-KD and *PGM*-KD IRSs may also have this component since iesRNA production may indirectly depend on scnRNAs, and since the excisase may use both scnRNAs and iesRNAs to recognize IESs. Given the substantial variation in retention scores between IESs in the *DCL2/3* cosilencing, we wondered if there might be a quantitative relationship between these scores and scnRNAs. In contrast to iesRNAs, whose densities increase substantially with *DCL5*-KD IRSs (Figure 5D), as IESs become increasingly sensitive to *DCL2/3* cosilencing (*DCL2/*3-KD IRS increases), on average the density of their scnRNAs remains the same (Figure 5A). We therefore suggest that the sensitivity of IESs to the *DCL2/3* cosilencing is not primarily due to the scnRNAs targeting them, and consequently that a similar amount of epigenetic control is being exerted upon all the epigenetic IESs, but that their sensitivity to this control is modulated by their underlying genetic properties. We also suggest that though there is greater variation in iesRNA density between IESs relative to the *DCL5*-KD IRSs, iesRNA-dependent IESs are also generally strongly influenced by their genetic properties (as is most apparent in Figure 4E–J).

### Evolution of IESs constrained by their excisase and sRNA targeting mechanisms

sRNA-based genome-defense mechanisms, conserved throughout eukaryotic evolution, are responsible for silencing transposons through heterochromatin formation (71). In *Paramecium* scnRNAs derived from these genome-defense mechanisms now serve both in battle and in peace, i.e. they have a defensive role, in targeting and elimination of foreign transposons, and a housekeeping role, guiding cleaning up the now resident, interstitial DNA which is no longer recognizable as transposons. Housekeeping (or error prevention) is likely the primary role of iesRNAs (18).

The proposed transposon origin of IESs in *Paramecium* (72) was recently bolstered by the finding of IESs which are clearly derived from transposons (37). When IESs originate as transposons they are likely to be long and have suboptimal end sequences for efficient recognition and removal by the IES excisase complex. The need to recognize and eliminate such long, suboptimal sequences (as epiIESs) may be fulfilled by scnRNAs. Other sequences which cannot be removed by the excisase and are located in critical genomic regions, such as coding sequences, will likely be deleterious to their host, and so we are unlikely to observe them. We propose that, due to the excisase preferences, over time selection weeds out longer IESs and IESs with end sequences which are difficult to recognize/excise, giving rise to the current IES length distribution and associated IES end-base preferences. Thus IESs will tend to evolve to become less dependent on epigenetic control systems. However, persistent genomic assault by mobile elements and mutations in existing IESs that result in reversion to suboptimal end sequences ensure the continued necessity of targeting by both scnRNAs and iesRNAs.

### Reconsidering epigenetic control of DNA deletion in *Tetrahymena thermophila*

To date, in contrast to the situation in *P. tetraurelia*, no nonepigenetically controlled IES deletion in *Tetrahymena thermophila* has been reported (55). Knockout of *Tetrahymena*'s scnRNA processing/binding Piwi-like protein, Twi1p, led to complete elimination of the multi-copy IESs that were examined (19), suggesting that none of these IES copies may be excised by *Tetrahymena*'s PiggyBac-like transposase alone. Furthermore, the ends of most *Tetrahymena* IESs are not precisely defined, and these IESs are typically not unique, unlike those of *Paramecium* (55). Thus it may neither be straightforward to tease apart the relative genetic and epigenetic contribution to IES excision in *Tetrahymena* using the approaches we have developed here, nor may we extend the inferences we have made in this paper directly to *Tetrahymena*. However, we believe that chromosome breakage during macronuclear development, a process related to IES deletion (19,55), is well-suited to these types of analyses because it appears to be subject to both genetic and epigenetic control, for the following reasons: (i) chromosome breakage sites in *Tetrahymena* have a specific 15 bp motif (73), indicating genetic control of this process; (ii) knockout of Twi1p, led to severe, but not complete, inhibition of chromosome breakage (19), suggesting that epigenetic control is also important for this process.

In conclusion, building upon a rich heritage of studies of genetic and epigenetic control in *Paramecium* (74), we now have the means to study and experimentally manipulate epigenetic control of DNA deletion and observe its genetic interplay on a genomic scale, which will enable detailed mechanistic studies of these processes. In the near future, it will be crucial to study the properties of the *Paramecium* IES excisase complex itself and how scnRNAs and iesRNAs guide this complex to DNA that should be deleted.

## SUPPLEMENTARY DATA

Supplementary Data are available at NAR Online.

SUPPLEMENTARY DATA
