# Supplementary material for: Genome-wide analysis of genetic and epigenetic control of programmed DNA deletion
Source: Nucleic Acids Res. 2014 Jul 12;42(14):8970–83. doi: 10.1093/nar/gku619 (PMC4132734; doi:10.1093/nar/gku619)
Supplement: SUPPLEMENTARY DATA [file supp_42_14_8970__index.html]

Genome-wide analysis of genetic and epigenetic control of programmed DNA deletion — Genome-wide analysis of genetic and epigenetic control of programmed DNA deletion — SUPPLEMENTARY DATA 

# Genome-wide analysis of genetic and epigenetic control of programmed DNA deletion

## SUPPLEMENTARY DATA

**Files in this Data Supplement:**

- SUPPLEMENTARY DATA
- SUPPLEMENTARY DATA
