# Supplementary material for: Genome-wide analysis of genetic and epigenetic control of programmed DNA deletion
Source: Nucleic Acids Res. 2014 Jul 12;42(14):8970–83. doi: 10.1093/nar/gku619 (PMC4132734; doi:10.1093/nar/gku619)
Supplement: SUPPLEMENTARY DATA [file supp_gku619_nar-01087-v-2014-File007.pdf]

Contents:

Supplementary figures - Figure S1-S3 (pages 1-2)

Supplementary tables - Table S1 (page 3)

Supplementary references (page 4)

SUPPLEMENTARY FIGURES

Figure S1. Correlations of IES retention scores between adjacent IESs. (A-C) Linear regressions of *PGM*-KD retention scores of adjacent IESs. Pearson's r and Spearman's r (r_s) are given above each graph. **(D)** Linear regressions of *PGM*-KD IRSs of IESs situated 10 IESs apart with > 10 kb of DNA sequence between them. **(E)** Linear regressions of *PGM*-KD IRSs of IES pairs < 200 bp of intervening macronuclear DNA sequence. **(F)** Linear regressions of *PGM*-KD IRS for randomly sampled pairs of IESs. **(G-H)** Change of correlation coefficients (Pearson's r) with distance between IESs; on the x-axis each data point is the correlation coefficient calculated for intervening distances between IESs less than the distance at that point.

Figure S2. Relationships between IES retention scores and end base frequencies. (A) Mean IES length vs. retention scores for Figure 3D are shown along with ewma lines plotted for data within 2 standard deviations (dotted vertical lines) of the mean retention score (dashed vertical line). **(B-E)** Base frequencies of positions 1-3 after the TA repeat compared to the IRS of *DCL5*-KD (B and C) and the control (D and E), for IESs from the first (26-36 bp) and third (45-55 bp) length peak. Only 45-55 bp IESs are shown, but similar trends are visible for ~10 bp windows surrounding longer IES length peaks. Lines are the exponentially-weighted mean averages (EWMA) with spans of 10 data points (intervals of 0.01) and are only plotted for data within 2 standard deviations (dotted vertical lines) of the mean retention score (dashed vertical line). **(F)** IES retention score distributions for *DCL2/3*-KD for common combinations of bases at IES ends internal to TA repeats, for IESs > 45 bp. To examine the distribution tails the y-axis is broken at *DCL2/3*-KD IRS 0.15 and 0.6.

Figure S3. Effects of gene silencing on sRNA densities and quantities. Headings above the subgraphs indicate the experimental condition or control, along with any additional constraints, and continue down the subgraph columns until the next heading. **(A-E)** Densities of IES-matching 25 nt scnRNAs and 25 nt iesRNAs (sRNAs/base) are the median values over IES retention intervals of 0.01 in the control, late development experiment. EWMA lines for spans of 5 data points (intervals of 0.01) are plotted for data within 2 standard deviations (dotted vertical lines) of the mean IES retention score (dashed vertical line) of the analyzed subset of IESs; since data outside 2 standard deviations is very limited and not very accurate trend lines beyond these intervals are not shown. A few isolated sRNA density outliers (> 0.4) are not shown for some of the subgraphs. **(D, E)** Graphs as in (A-C), but with quantities of scnRNAs vs *DCL2/3*-KD IRS and iesRNAs vs *DCL5*-KD IRS, respectively. **(F, G)** scnRNA and iesRNA quantities vs IES lengths. EWMA lines draw for spans of 20 bp are are plotted for data within 2 standard deviations (dotted vertical lines) of the mean IES length.

SUPPLEMENTARY TABLES

Table S1

Historical classification	Genbank name	Reference ID	Maternal control score[(iii)]	*DCL2/3*-KD IRS	*DCL5*-KD IRS	*PGM*-KD IRS	Control IRS
mc	51A2591	IESPGM.PTET51.1.106.284913	0.99	0.54	0.00	0.91	0.00
mc	51G4404	IESPGM.PTET51.1.51.455615	0.97	0.60	0.01	0.84	0.00
mc	51G2832	IESPGM.PTET51.1.51.454043	0.87	0.23	0.00	0.77	0.01
mc	51A6649	IESPGM.PTET51.1.106.288995	0.58	0.56	0.01	0.81	0.00
mc	51A-712	IESPGM.PTET51.1.106.281631	0.34	0.07	0.06	0.78	0.00
mc	51G-11[(i)]	IESPGM.PTET51.1.51.451201	ND	0.05	0.20	0.80	0.00
mc	MT[(ii)]	Not annotated	ND	ND	ND	ND	ND
non-mc	51G1832	IESPGM.PTET51.1.51.453043	0.00	0.00	0.00	0.73	0.00
non-mc	51G1413	IESPGM.PTET51.1.51.452624	0.00	0.01	0.02	0.78	0.00
non-mc	51A6435	IESPGM.PTET51.1.106.288781	0.00	0.00	0.00	0.77	0.00
non-mc	51A4578	IESPGM.PTET51.1.106.286924	0.00	0.05	0.00	0.78	0.00
non-mc	51A4404	IESPGM.PTET51.1.106.286750	0.00	0.00	0.00	0.83	0.00
non-mc	51A1835	IESPGM.PTET51.1.106.284157	0.00	0.00	0.00	0.80	0.00
non-mc	51A1416	IESPGM.PTET51.1.106.283738	0.00	0.00	0.00	0.76	0.00
non-mc	51A-10	IESPGM.PTET51.1.106.282313	0.00	0.00	0.00	0.87	0.00

(i) The maternal control status of this IES is known but unpublished (5).

(ii) No IES+ and IES- reads were determined for this, since this mating type IES (MT) was not annotated in the genome assembly used. This "IES" is present in the macronuclear genome of the sequenced strain (whereas in other strains it may be absent (6)).

(iii) Maternal control scores were obtained from the maximal retention observed in Figure 6 of (7)

ND = Not determined.

The historical maternal control classification of IESs other than those in (i) and (ii) was determined in (7,8).

SUPPLEMENTARY REFERENCES

1. Arnaiz, O. and Sperling, L. (2011) ParameciumDB in 2011: new tools and new data for functional and comparative genomics of the model ciliate Paramecium tetraurelia. *Nucleic acids research*, **39**, D632-636.
2. Edgar, R.C. (2004) MUSCLE: multiple sequence alignment with high accuracy and high throughput. *Nucleic acids research*, **32**, 1792-1797.
3. Wheeler, T.J., Clements, J. and Finn, R.D. (2014) Skylign: a tool for creating informative, interactive logos representing sequence alignments and profile hidden Markov models. *BMC bioinformatics*, **15**, 7.

Figure S1

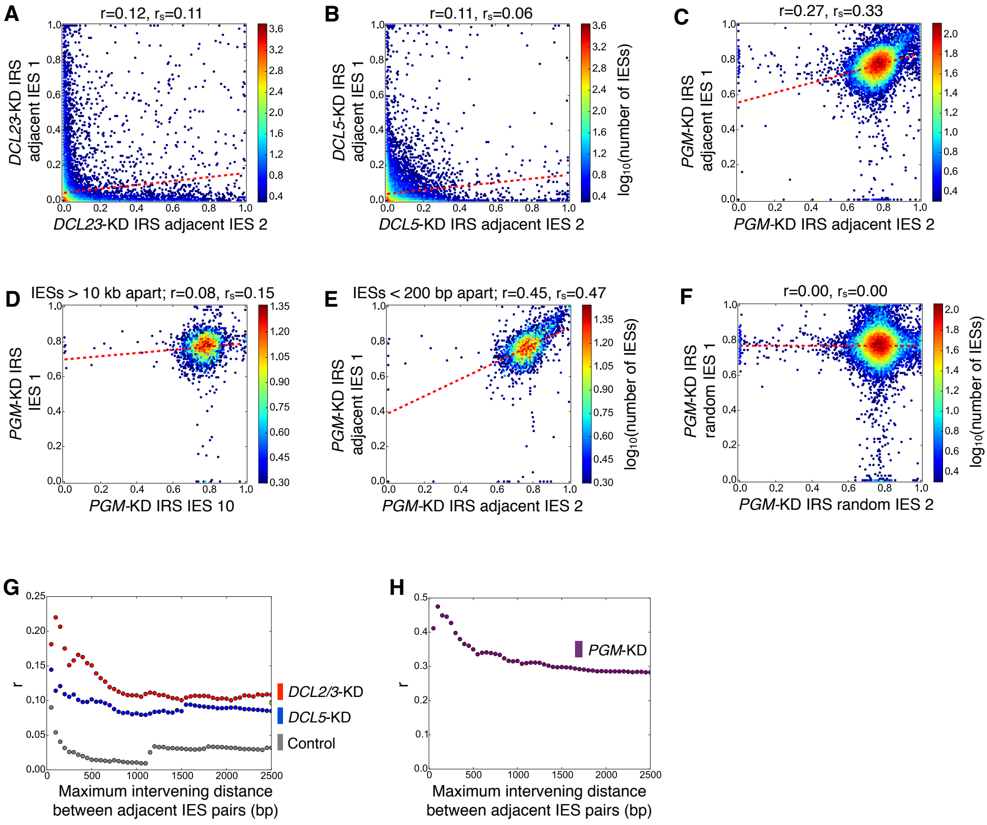

A
r=0.12, rs=0.11
DCL23-KD IRS adjacent IES 1
DCL23-KD IRS adjacent IES 2
B
r=0.11, rs=0.06
DCL5-KD IRS adjacent IES 1
DCL5-KD IRS adjacent IES 2
log10(number of IESs)
C
r=0.27, rs=0.33
PGM-KD IRS adjacent IES 1
PGM-KD IRS adjacent IES 2
D
IESs > 10 kb apart; r=0.08, rs=0.15
PGM-KD IRS IES 1
PGM-KD IRS IES 10
E
IESs < 200 bp apart; r=0.45, rs=0.47
PGM-KD IRS adjacent IES 1
PGM-KD IRS adjacent IES 2
log10(number of IESs)
F
r=0.00, rs=0.00
PGM-KD IRS random IES 1
PGM-KD IRS random IES 2
log10(number of IESs)
G
r
Maximum intervening distance between adjacent IES pairs (bp)
DCL2/3-KD
DCL5-KD
Control
H
r
PGM-KD
Maximum intervening distance between adjacent IES pairs (bp)

Figure S2

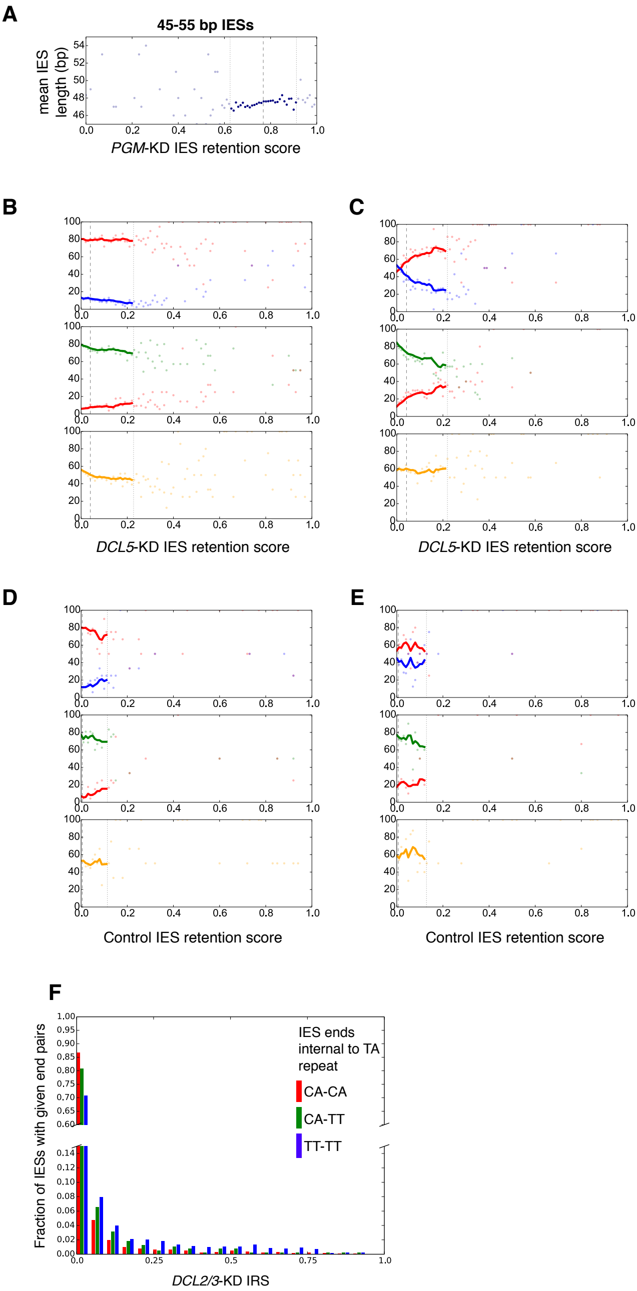

A
45-55 bp IESs
mean IES length (bp)
PGM-KD IES retention score
B
DCL5-KD IES retention score
C
DCL5-KD IES retention score
D
Control IES retention score
E
Control IES retention score
F
Fraction of IESs with given end pairs
IES ends internal to TA repeat
CA-CA
CA-TT
TT-TT
DCL2/3-KD IRS

Figure S3

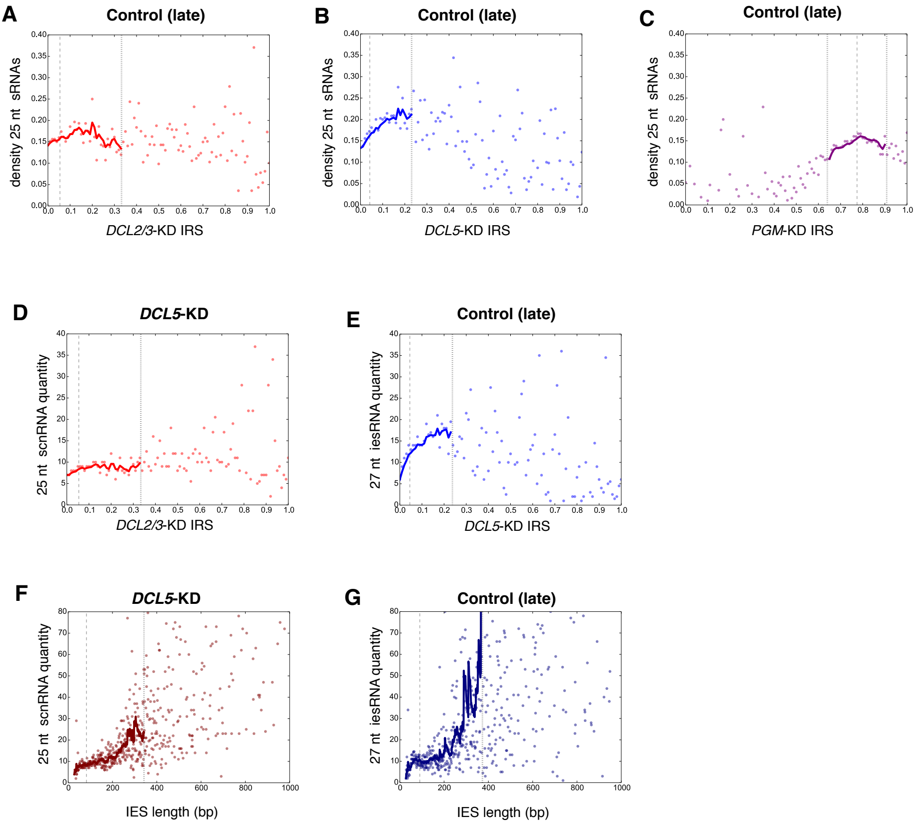

A
Control (late)
density 25 nt sRNAs
DCL2/3-KD IRS
B
Control (late)
density 25 nt sRNAs
DCL5-KD IRS
C
Control (late)
density 25 nt sRNAs
PGM-KD IRS
D
DCL5-KD
25 nt scnRNA quantity
DCL2/3-KD IRS
E
Control (late)
27 nt iesRNA quantity
DCL5-KD IRS
F
DCL5-KD
25 nt scnRNA quantity
IES length (bp)
G
Control (late)
27 nt iesRNA quantity
IES length (bp)
